# Tubulointerstitial nephritis and uveitis syndrome in a patient with diabetic nephropathy: a case report and literature review

**DOI:** 10.3389/fmed.2026.1810535

**Published:** 2026-07-29

**Authors:** Dan-Fang Deng, La-Mei Lin, Lan Wang, Chang-Jiang Wang, Zhong-Ping Wei, Yan-Ping Wu, Xiao-Qin Wang, Xin-Rong Zou

**Affiliations:** 1 Department of Nephrology, Hubei Provincial Hospital of Traditional Chinese Medicine, Wuhan, China; 2 Hubei Key Laboratory of Theory and Application Research of Liver and Kidney in Traditional Chinese Medicine, Affiliated Hospital of Hubei University of Traditional Chinese Medicine, Wuhan, China; 3 Hubei Shizhen Laboratory, Wuhan, China; 4 Hubei Institute of Traditional Chinese Medicine, Wuhan, China

**Keywords:** diabetic nephropathy, interstitial, nephritis, tubulointerstitial nephritis and uveitis syndrome, uveitis

## Abstract

**Background:**

The coexistence of tubulointerstitial nephritis and uveitis (TINU) syndrome with diabetic nephropathy is an uncommon clinical occurrence and poses diagnostic challenges.

**Case presentation:**

A 63-year-old female patient presented with ocular pain, conjunctival congestion, blurred vision, and photophobia. These manifestations were followed by a rapid deterioration of renal function. Renal biopsy revealed histopathological features consistent diabetic nephropathy, including interstitial nephritis and acute tubular injury. Ophthalmologic evaluation demonstrated bilateral anterior uveitis. After exclusion of other primary etiologies, a diagnosis of TINU syndrome complicated by diabetic nephropathy was established. Following treatment with glucocorticoids, ocular symptoms, renal dysfunction, and systemic inflammatory markers demonstrated marked improvement.

**Conclusion:**

TINU syndrome should be considered in individuals with diabetic nephropathy who develop acute kidney injury and concurrent uveitis. Early diagnosis through histopathological evaluation and multidisciplinary management are critical for improving clinical outcomes.

## 1 Introduction

Tubulointerstitial nephritis and uveitis (TINU) syndrome is a rare cause of acute tubulointerstitial nephritis and is frequently misdiagnosed as drug-induced interstitial nephritis. Acute tubulointerstitial nephritis represents one of the most common causes of acute kidney injury (AKI), accounting for approximately 15% to 27% of renal biopsy-confirmed AKI cases (1). TINU syndrome is characterized by nonspecific systemic manifestations, and renal and ocular symptoms often occur asynchronously, which contributes to misdiagnosis or delayed recognition. Since the initial description of TINU syndrome in 1975, approximately 600 cases have been reported worldwide (2). Currently, no standardized international or national treatment guidelines exist; however, most patients receive systemic and or topical glucocorticoid therapy. TINU syndrome was previously considered to be associated with a favorable renal prognosis; however, emerging evidence indicates that a substantial proportion of individuals experience incomplete recovery of renal function, largely attributable to delayed diagnosis and treatment (3, 4). The present report describes a patient with TINU syndrome complicated by diabetic nephropathy, with the objective of increasing clinical awareness of this uncommon disease entity.

## 2 Case presentation

A 63-year-old female patient was admitted to the nephrology department on June 20, 2023, with facial and lower limb edema accompanied by intermittent fever persisting for 3 weeks. No examinations or treatments had been performed before June 8, 2023. On that date, the patient presented to the ophthalmology department because of bilateral ocular congestion, eye pain, photophobia, and blurred vision. Laboratory testing demonstrated a serum creatinine (SCr) level of 169 μmol/L and a fasting blood glucose level of 26.7 mmol/L. The patient was subsequently admitted to the endocrinology department and diagnosed with type 2 diabetes mellitus with poor glycemic control, type 2 diabetic kidney disease, and renal insufficiency.

Despite treatment with hypoglycemic agents, antibiotics, antipyretics, and renal protective therapy, the patient's edema and fever persisted, urine output progressively decreased, and SCr levels continued to increase. On June 17, 2023, SCr reached 262 μmol/L, and the patient was transferred to the nephrology department on June 20, 2023. At the time of admission, the patient presented with facial and bilateral lower limb edema, nocturia, conjunctival congestion, poor appetite, occasional nausea and vomiting, reduced urine output, constipation, and a reported weight gain of approximately 5 kg over the preceding 2 weeks. The medical history included type 2 diabetes for 14 years and hypertension for 10 years. Cervical lymph node dissection had been performed in 2020, and pathological examination demonstrated granulomatous inflammation with necrosis. No history of drug or food allergies was reported, and there was no relevant family history.

On physical examination, vital signs were as follows: body temperature 36.8 °C, respiratory rate 18 breaths per minute, pulse rate 74 beats per minute, and blood pressure 163/97 mmHg. Conjunctival congestion with secretions was observed. Enlarged lymph nodes were palpable in the supraclavicular and inguinal regions, with the largest measuring approximately 3 cm × 2 cm, exhibiting a firm texture, clear boundaries, and absence of tenderness. Pulmonary auscultation revealed slightly coarse breath sounds bilaterally. Cardiac examination demonstrated a mildly enlarged left cardiac border with a regular rhythm, and no murmurs were detected in any valve auscultation area. Abdominal examination revealed no remarkable findings. Pitting edema was present in both lower extremities. Skin ulcers with purulent secretions were noted in the sacrococcygeal region.

**Figure 1:**
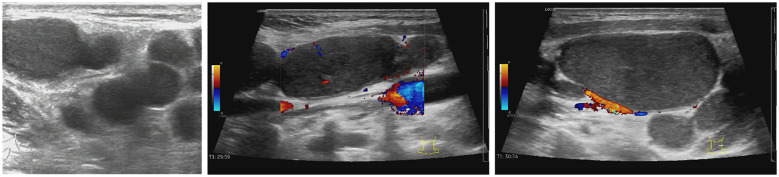
Color Doppler ultrasonography of cervical lymph node biopsy and associated vascular flow.

**Figure 2:**
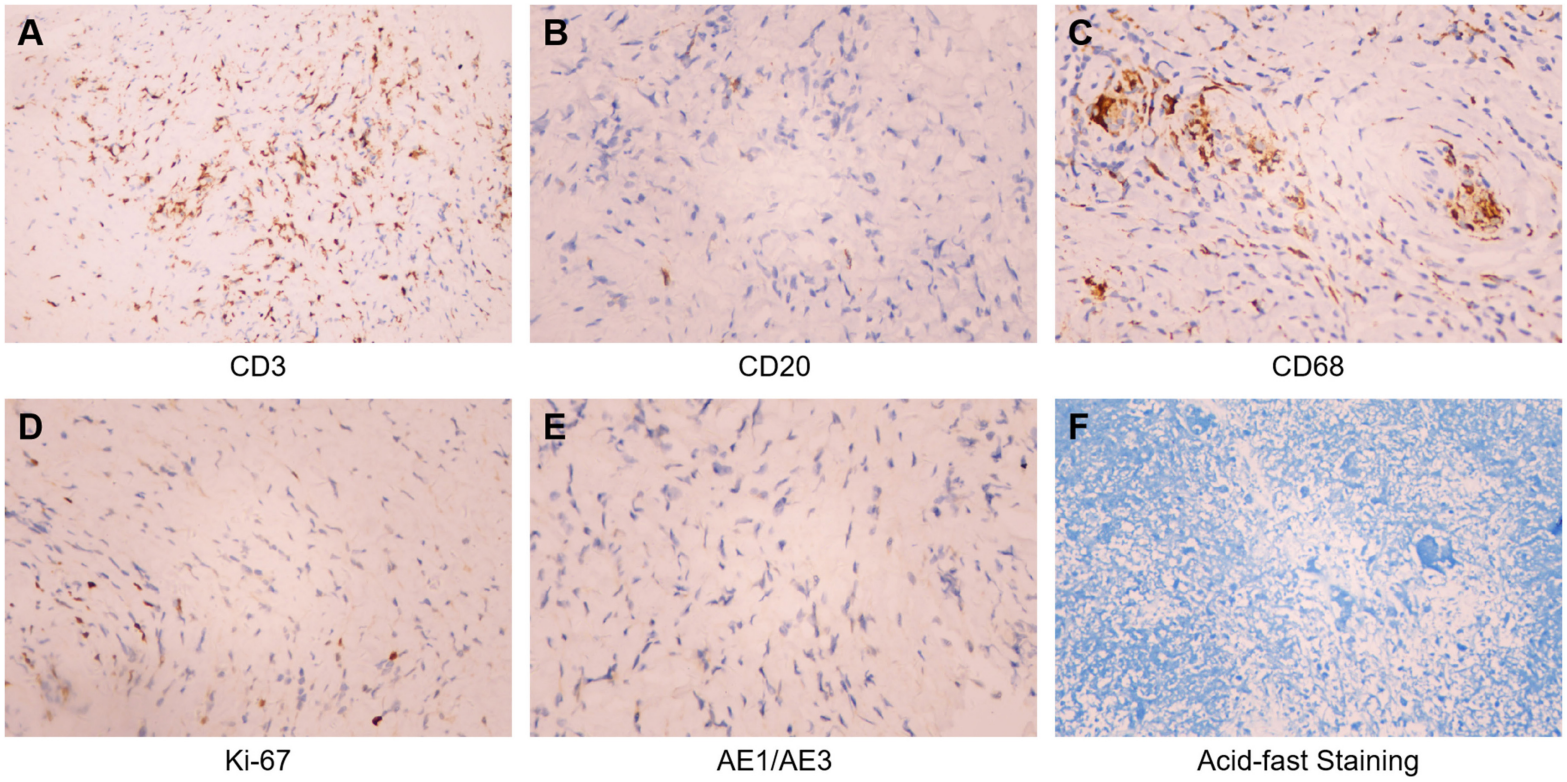
Histopathological and immunohistochemical findings of cervical lymph node biopsy. Immunohistochemical staining for **(A)** CD3 (×400), **(B)** CD20 (×400), **(C)** CD68 (×400), **(D)** Ki-67 (×400), and **(E)** AE1/AE3 (×400); **(F)** acid fast staining (×400).

Initial laboratory results (Table 1) demonstrated anemia (hemoglobin 76 g/L), eosinophilia (eosinophils 17.5%), marked inflammatory response (C-reactive protein 205.29 mg/L, procalcitonin 3.32 ng/ml, erythrocyte sedimentation rate 73.0 mm/h). Liver function parameters were abnormal (γ-glutamyl transferase 181 U/L, alkaline phosphatase 542 U/L, lactate dehydrogenase 361 U/L). Serum β2-microglobulin was markedly elevated (23.12 mg/L), and urinalysis revealed hematuria (2+), urinary protein 2+, urinary glucose 3+, and urinary β2-microglobulin concentration of 176.92 mg/L. SCr was 444 μmol/L. Electrocardiography findings were normal. Pulmonary computed tomography demonstrated infectious lesions in both lungs with small amounts of pleural effusion. Color Renal ultrasonography revealed normal renal morphology, size, and vascular flow. Multiple enlarged lymph nodes were detected bilaterally, some with hilar vascularity; the largest measured 3.3 cm × 1.5 cm in the right Zone III and 4.2 cm × 2.0 cm in the left Zone IV, indicating a possible lymphoproliferative disorder (Figure 1). A lymph node biopsy was therefore performed. Histopathological examination revealed coagulative necrosis and multinucleated giant cells. Acid-fast bacilli were not detected by staining. Immunohistochemical analysis demonstrated positivity for CD3, CD20, CD68, and Ki-67 (<5%), with negativity for AE1/AE3, thereby excluding tuberculous lymphadenitis and lymphoma (Figure 2).

**Table 1:** Baseline laboratory findings at hospital admission.

Complete blood counts	Observed value	Reference range	Biochemistry	Observed value	Reference range
WBC	5.4	3.5–9.9^*^ 10^9^/L	TP	56.7	65–85 g/L
GRAN	90.9	40–75%	Albumin	24.0	40–55 g/L
EO	17.50	0.4–8%	AST	33	7–40 U/L
RBC	2.88	3.8–5.1^*^ 10^12^/L	ALT	43	13–35 U/L
HGB	76 g/L	115–150 g/L	γ-GGT	181	7–45 U/L
RET	4.04	0.76–2.21%	ALP	542	50–135 U/L
			LDH	361	120–250 U/L
Urinalysis			β2–MG	23.12	1.0–3.0 mg/L
BLD	2+	–	BUN	27.4	3.1–8.8 mmol/L
PRO	2+	–	UA	589	155–357 μmol/L
Glucose	3+	–	SCr	444	41–81 μmol/L
β2–MG	176.92	0.1–0.3 mg/L	eGFR	9.0	>90 ml/min/1.73 m^2^)
UACR	196	0–30 mg/g	Sodium	134	137–147 mmol/L
RBP	14.14	<0.7 mg/L	Potassium	4.04	3.5–5.3 mmol/L
24 h–PRO	818.5	<150 mg/24hr	Chloride	92.2	99–110 mmol/L
			Calcium	2.2	2.11–2.52mmol/L
Inflammation			Phosphorus	1.87	0.85–1.51mmol/L
CRP	205.29	0–4 mg/L	Magnesium	0.84	0.75–1.02mmol/L
PCT	3.32	0–0.06 ng/ml	NT–proBNP	6771	0–900 pg/ml
ESR	73	0–20 mm/h	HbA1C	13.3	4.0–6.0%
Immunology			Others		
ANA	1:320	–	FT3	1.73	2.8–7.1 pmol/L
ENA	–	–	FT4	10.5	9–25 pmol/L
ANCA	–	–	TSH	1.2	0.3–5 mIU/L
PLA2R	1.67	<20 RU/ml	HBsAg	–	–
GBM	–	–	HCV–cAg	–	–
dRVVT–SR	1.48	<1.2	HIV	–	–
Immunoglobulin electrophoresis	–	–	Torch Anti–RVIgG	3.16	0–2 AU/ml
C3	0.99	0.7–1.4 g/L	Torch CMVIgG	4.68	0–2 AU/ml
C4	0.32	0.1–0.4 g/L	Torch HSVIgG	12.6	0–2 AU/ml
IgG	14.76	8.6–17.4 g/L	Blood culture	–	–
IgG4	0.61	0.03–2.01g/L	Ulcer secretions	Escherichia coli	–
IgA	1.92	1.0–4.2 g/L	Coombs	+	–
IgM	1.3	0.5–2.8 g/L	Tuberculosis bacillus γ-interferon release test	–	–

Ophthalmologic evaluation revealed marked bilateral eyelid erythema, ciliary congestion, and conjunctival edema. A large amount of secretion was observed in the conjunctival sac, accompanied by distinct striated corneal endothelial opacity, vitreous opacity, and blurred visualization of the fundus. Fluorescein angiography demonstrated patchy retinal fluorescein leakage in the early phase and diffuse fluorescein leakage in the late phase, findings consistent with uveitis. Based on medical history and auxiliary examinations, a preliminary diagnosis of uveitis combined with acute kidney injury was considered. To further clarify the etiology, a renal biopsy was performed.

**Figure 3:**
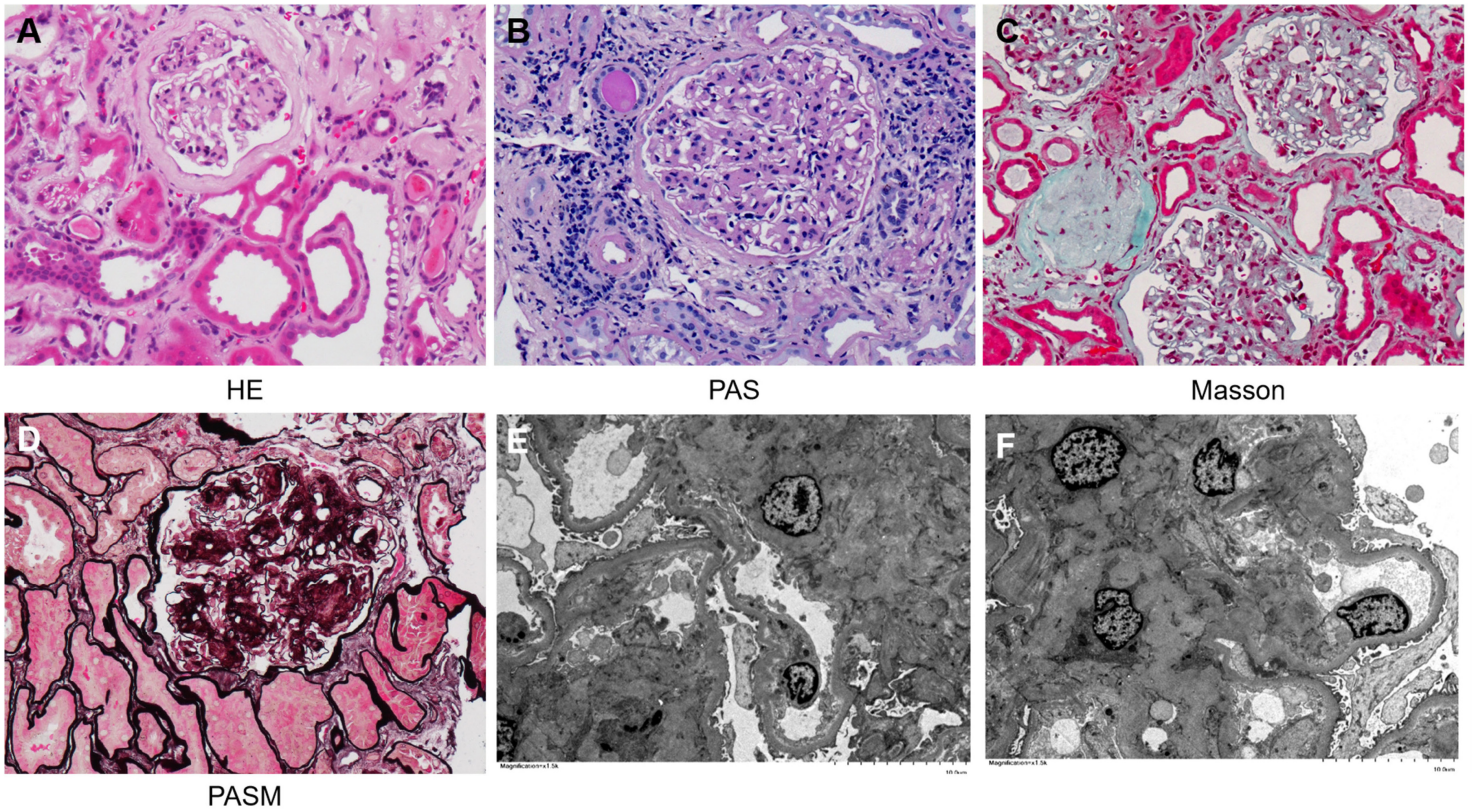
Renal biopsy findings in a patient with diabetic nephropathy complicated by tubulointerstitial nephritis and uveitis syndrome. **(A)** Hematoxylin and eosin staining (×200), **(B)** periodic acid Schiff staining (×200), **(C)** Masson trichrome staining (×200), **(D)** periodic acid silver methenamine staining (×200), and **(E, F)** electron microscopy (×5,000).

Light microscopy of the renal tissue demonstrated that 9 of 21 glomeruli demonstrated global sclerosis and 3 of 21 glomeruli exhibited segmental sclerosis. Mild to moderate mesangial expansion was observed, with severe segmental expansion and the presence of Kimmelstiel–Wilson nodules. The tubulointerstitial compartment demonstrated severe acute lesions with patchy loss of tubular brush borders. Chronic changes were mild and included focal tubular atrophy, thickened tubular basement membranes, protein casts, patchy infiltration of mononuclear cells and plasma cells, and occasional eosinophils (Figures 3A–D). Immunofluorescence was negative for immunoglobulin G, A, M; complement components C3 and C1q, light chains κ, λ, and fibrinogen. Electron microscopy demonstrated diffuse thickening of the glomerular basement membranes without electron-dense deposits. Some podocytes demonstrated vacuolar degeneration with fusion of foot processes. Mesangial cells and matrix exhibited moderate expansion without electron-dense deposits, and partial thickening of the tubular basement membranes was observed. Minimal collagen fiber deposition was present in the renal interstitium (Figures 3E, F). These findings supported a pathological diagnosis of diabetic nephropathy with severe acute interstitial nephritis and mild chronic tubulointerstitial changes.

Based on the clinical manifestations, laboratory and imaging findings, renal biopsy results, and ophthalmologic evaluation, differential diagnoses including lymph node tuberculosis, lymphoma, and systemic lupus erythematosus were excluded. A final diagnosis of diabetic nephropathy complicated by tubulointerstitial nephritis and uveitis syndrome was established. Blood glucose levels were closely monitored, and topical ocular glucocorticoid therapy was initiated.

Systemic treatment commenced on July 1, 2023, with intravenous methylprednisolone at a dose of 40 mg/day for three consecutive days. This intervention was associated with a marked reduction in cervical lymph node enlargement and a decrease in SCr to 358 μmol/L. The regimen was followed by oral methylprednisolone at 32 mg/day for 3 days and subsequently 20 mg/day for 5 days. After 3 weeks of comprehensive treatment, body temperature normalized, edema resolved, ocular symptoms disappeared, and enlarged cervical lymph nodes were no longer palpable. SCr decreased to 180 μmol/L, and the glucocorticoid dose was gradually tapered and discontinued in September 2023.

**Figure 4:**
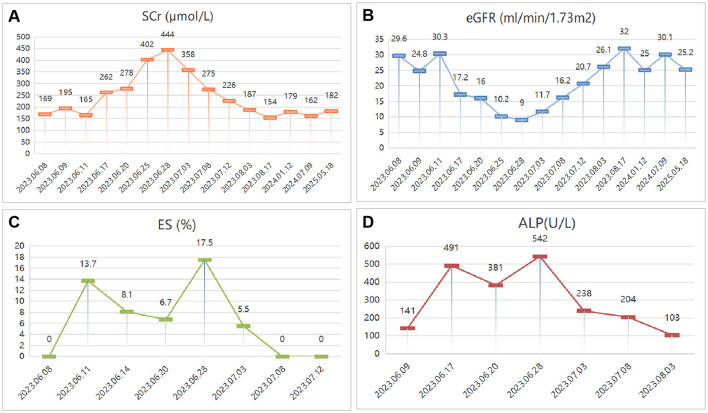
Longitudinal changes in serum indicators during glucocorticoid therapy. **(A)** Serum creatinine; **(B)** Estimated glomerular filtration rate; **(C)** Eosinophil percentage; **(D)** Alkaline phosphatase.

During a follow-up period of nearly 2 years, uveitis did not recur; however, SCr levels fluctuated between 150 μmol/L and 180 μmol/L. Changes in relevant clinical and laboratory parameters during the course of glucocorticoid therapy are presented in Figure 4.

## 3 Discussion

TINU syndrome is a multisystem autoimmune disorder characterized primarily by acute tubulointerstitial nephritis accompanied by uveitis and involves both humoral and cellular immune mechanisms (5). In 1975, Dobrin et al. first described this condition in two cases presenting with anterior uveitis, acute eosinophilic interstitial nephritis, and bone marrow granulomas. In 1985, Vanhaesebrouck introduced the term “TINU syndrome” and defined it as a triad consisting of systemic inflammatory manifestations, tubulointerstitial nephritis, and uveitis, with the clinical and pathological features of all three components considered essential for diagnosis (6). As a rare autoimmune disease with global sporadic distribution, regional case reports from Latin America have further enriched the clinical understanding of TINU syndrome. Pinheiro et al. (7) documented the first formally reported case of TINU syndrome in Brazil, filling the regional epidemiological gap in South America Subsequently, Montejo-Hernández et al. (8) supplemented the clinical data of TINU syndrome in Colombia through a comprehensive case report and literature review, further demonstrating the phenotypic heterogeneity and diagnostic challenges of this disorder in Latin American populations. These regional landmark studies complement the global case spectrum of TINU syndrome and provide valuable comparative references for atypical clinical presentations of the disease.

The etiology of TINU syndrome remains incompletely understood. Preceding infections caused by pathogens such as *Chlamydia spp., Toxoplasma gondii*, and Epstein–Barr virus have been reported as potential triggers. In addition, exposure to nonsteroidal anti-inflammatory drugs, antibiotics, and other medications has been associated with disease onset. Immune dysregulation appears to play a central role in pathogenesis. The predominance of T lymphocytes in early disease stages supports a cell mediated autoimmune mechanism as the primary driver, whereas the detection of immune complex deposition in later stages indicates that humoral immunity may contribute as a secondary event in disease progression (9, 10). TINU syndrome has also been linked to the human leukocyte antigen (HLA) system, with certain alleles considered genetic susceptibility factors. Increased frequencies of high risk HLA genotypes, including HLA A2, HLA A24, and HLA DQA1, have been reported in affected patients, indicating an underlying genetic predisposition (11, 12).

Clinically, TINU syndrome is characterized by sequential or simultaneous renal or ocular involvement, often accompanied by nonspecific systemic symptoms. The presentation varies substantially between individuals (Table 2). Initial presentation may involve ocular symptoms such as uveitis, or renal involvement manifesting as acute interstitial damage (13). Whether concurrent renal and ocular involvement reflects distinct manifestations of a shared immune mechanism or independent immune targets remains unclear.

**Table 2:** Clinical manifestations of tubulointerstitial nephritis and uveitis in tubulointerstitial nephritis and uveitis syndrome.

Acute interstitial nephritis (AIN)	Uveitis
Fever	Eye pain
Rash	Redness
Fatigue	Itching
Anorexia	Dry eyes
Weight loss	Photophobia
Abdominal or flank pain	Tearing
Headache	Decreased vision
Arthralgia	Foreign body sensation
Myalgia	Floaters
Osphyalgia	Metamorphopsia
Nocturia	Eyelid edema
Polyuria	Conjunctival injection
Facial edema	Ciliary injection
Generalized lymphadenopathy	Keratic precipitates
Anemia	Turbidity of aqueous humor
Eosinophilia	Vitreous opacity
Abnormal urination (gross or microscopic hematuria, mild proteinuria, aseptic leukocyturia)	Macular edema
Abnormal liver function	Optic disc swelling
Abnormal renal function	Retinal edema
Elevated β-2 microglobulin (both in the serum and urine)	Retinal vasculitis
Elevated inflammatory parameters	Chorioretinitis
Tubulointerstitial inflammation	Chorioretinal lesions

In 2001, Mandeville et al. proposed a diagnostic framework to improve the accuracy of TINU syndrome identification based on clinical manifestations and ancillary test results (Supplementary Figure 1) (14). More recently, the Standardization of Uveitis Nomenclature (SUN) Working Group proposed classification criteria for TINU syndrome (Supplementary Figure 1) (15). According to these criteria, a diagnosis of TINU syndrome requires the presence of anterior uveitis and tubulointerstitial nephritis, with nephritis confirmed by renal biopsy or abnormal renal function tests, after the exclusion of alternative etiologies such as syphilis and sarcoidosis (5, 15).

In the present case, the patient developed rapidly progressive renal dysfunction consistent with AKI, accompanied by mild albuminuria and markedly elevated β2-microglobulin levels in both blood and urine. Renal biopsy findings, together with comprehensive laboratory evaluations, confirmed the presence of acute interstitial nephritis. Bilateral anterior uveitis represents the most common ophthalmic manifestation of TINU syndrome; however, involvement of the posterior uveal segment has also been described in multiple reports (16–18). In this case, the patient exhibited bilateral ocular pain, photophobia, tearing, and blurred vision, consistent with anterior uveitis. In addition, conjunctival ciliary congestion, vitreous opacities, and fluorescein angiographic findings including retinal capillary leakage and optic disc staining indicated posterior segment involvement (19, 20). Systemic manifestations were also evident, including fever, generalized lymphadenopathy, anemia, abnormal liver function, pronounced eosinophilia, elevated C-reactive protein levels, and elevated erythrocyte sedimentation rate. Collectively, these findings supported the diagnosis of TINU syndrome.

TINU syndrome remains a diagnosis of exclusion, and accurate identification relies on renal biopsy and comprehensive clinical evaluation to exclude alternative systemic conditions with overlapping features, such as Behçet disease, sarcoidosis, systemic lupus erythematosus, granulomatosis with polyangiitis, and Sjögren syndrome (5, 21). Diagnostic challenges are amplified when renal and ocular manifestations occur asynchronously. In the present case, ocular symptoms preceded the rapid decline in renal function, increasing the likelihood of delayed or missed diagnosis, particularly in the context of preexisting kidney disease. Deterioration of renal function in such settings is frequently attributed to progression of the underlying disease rather than the coexistence of superimposed TINU syndrome.

The presence of concurrent diabetic nephropathy further complicated diagnostic assessment. Renal biopsy demonstrated classical pathological features of diabetic nephropathy, including Kimmelstiel–Wilson nodules and mesangial matrix expansion, in addition to severe acute tubular injury and interstitial infiltration by mononuclear cells and plasma cells, consistent with TINU syndrome superimposed on diabetic nephropathy. Therefore, in patients with underlying chronic kidney disease who develop acute renal deterioration, careful clinical history taking, standardized laboratory assessment of blood and urine, and renal biopsy are essential to confirm concurrent acute interstitial nephritis and avoid underdiagnosis of TINU syndrome. Metabolic comorbidities such as type 2 diabetes and hypertension may further modify the immune microenvironment and increase susceptibility to disease. A national retrospective cohort study of 41 patients with biopsy-confirmed TINU syndrome demonstrated that most patients had preexisting comorbidities, including autoimmune disorders, metabolic diseases, and cardiovascular conditions, with diabetes and or hypertension present in 51% of cases (22). Chronic hyperglycemia may contribute to endothelial dysfunction and oxidative stress, thereby promoting tubulointerstitial inflammation, while hypertension-related alterations in renal hemodynamics may further exacerbate renal injury. Although these mechanisms provide a plausible pathological basis for TINU syndrome development in individuals with metabolic disease, the causal nature of these associations remains uncertain.

Although certain cases of TINU syndrome follow a self-limiting course, early and appropriate treatment is essential to promote recovery and reduce the risk of chronic complications (3). Glucocorticoids are currently regarded as first line therapy, with effects attributed to suppression of interstitial inflammatory cell infiltration, mitigation of tubular epithelial injury, and inhibition of immune mediated damage.

Considerable heterogeneity exists in reported glucocorticoid dosing regimens and treatment duration. Huang et al. analyzed 80 Chinese patients with TINU syndrome and reported that most received intravenous or oral glucocorticoids at doses ranging from 0.5 to 1 mg/kg/day, followed by gradual tapering over 6 to 8 weeks (23). Some patients underwent high dose short course or pulse therapy, with total treatment duration ranging from 14 to 207 weeks. In contrast, Legendre et al. reported that 85% of patients received oral systemic corticosteroids at doses of 0.5 to 1 mg/kg/day, with treatment durations ranging from 0.2 to 39 months, with a median duration of 8 months (22). Corticosteroid therapy was associated with improvement in renal function and lower recurrence rates of uveitis compared with untreated patients, although the degree of improvement was not significantly correlated with corticosteroid dosage. Despite responsiveness of both renal and ocular manifestations to corticosteroids, long-term follow-up studies indicate a risk of relapse, most commonly involving ocular inflammation, which often demonstrates a chronic and recurrent course in contrast to renal involvement (24, 25). Nevertheless, favorable visual outcomes with minimal complications are achievable with appropriate management strategies (26). Given the potential severity of relapse and sequelae, immunomodulatory therapy is often required (17).

In patients who are refractory to corticosteroids or demonstrate inadequate response, agents such as mycophenolate mofetil, azathioprine, cyclosporine, cyclophosphamide, and methotrexate have been utilized with varying degrees of success (8, 27–33). Limited reports have also described the use of biologic agents, including adalimumab, infliximab, and other tumor necrosis factor-alpha inhibitors, particularly in cases with insufficient response or intolerance to conventional immunomodulatory therapy (34, 35).

Renal involvement of TINU syndrome may resolve spontaneously or following corticosteroid therapy (14); however, complete recovery is not universal. Legendre et al. reported that 32% of patients developed moderate to severe chronic kidney disease after 1 year of follow-up despite corticosteroid treatment (22). A prospective cohort study from China reported that up to 92% of patients developed stage three to four chronic kidney disease and or tubular dysfunction 12 months after renal biopsy (36). Azathioprine and mycophenolate mofetil are generally preferred immunomodulatory agents for renal involvement, particularly in patients with advanced disease or corticosteroid resistance (37). However, the requirement for second line therapy has not been independently associated with the development of chronic kidney disease during follow-up (31). A small subset of patients progress to uremia and electrolyte disturbances requiring renal replacement therapy, even after immunomodulatory treatment (23, 31).

In the present case, multidisciplinary management involving ophthalmology, endocrinology, and infectious disease specialists enabled stepwise methylprednisolone therapy under strict glycemic monitoring, intensive insulin therapy, and dietary management. Blood glucose levels remained stable without severe hyperglycemia or ketoacidosis. Over a follow-up period of nearly 2 years, uveitis did not recur, while serum creatinine levels fluctuated between 150 and 180 μmol/L, indicating partial but incomplete recovery of renal function. These findings underscore that renal prognosis in TINU syndrome depends not only on effective control of inflammation but also on the extent of preexisting kidney damage. In patients with comorbid chronic kidney disease, corticosteroid therapy requires careful balancing of immunosuppression and renal protection to minimize infection risk. Long-term follow up is essential to monitor for uveitis recurrence and progression of diabetic kidney disease.

Both pediatric patients and adults are susceptible to TINU syndrome. Pediatric cases tend to experience more frequent ocular relapses and are less likely to develop acute kidney injury compared with adults (22, 31). Shi et al. reported follow up longer than 6 months in 51 patients and found that 20 patients developed recurrent renal disease or progressed to chronic kidney disease, with a significantly higher incidence among adults (58%) than children (18%) (38). This disparity may be attributed to reduced renal functional reserve in adults, possibly due to lifestyle factors and comorbidities such as hypercholesterolemia, hypertension, and overweight status, rendering adult kidneys more vulnerable to additional insults such as tubulointerstitial nephritis. However, evidence supporting this hypothesis remains limited.

Glucocorticoids remain the primary therapy for pediatric TINU syndrome. In the study by Shi et al., all patients younger than 18 years were treated with glucocorticoids alone (38). Caplas et al. described a 12 year old girl with atypical TINU syndrome who was treated with oral corticosteroids and mycophenolate mofetil, resulting in marked symptom resolution, although recurrent anterior segment inflammation during prednisone tapering necessitated escalation to multiple immunomodulatory agents, including adalimumab (39). Given the rarity of TINU syndrome, standardized, evidence based treatment guidelines are currently lacking. Therapeutic strategies regarding agent selection, dosage, and treatment duration should be individualized based on baseline renal function, ocular disease severity, and the presence of comorbidities. Long-term prospective studies are required to provide more robust evidence to guide clinical management.

## 4 Conclusions

This report describes a rare case of TINU syndrome complicated by diabetic nephropathy, a clinical scenario that is highly susceptible to misdiagnosis in the absence of careful diagnostic evaluation. For internists, endocrinologists, ophthalmologists and nephrologists, any unexplained uveitis or related inflammatory ocular manifestations in patients with renal dysfunction should alert clinicians to the possibility of TINU syndrome, and renal biopsy is strongly recommended to confirm the diagnosis. Therapeutic management requires a careful balance between the anti-inflammatory effects of glucocorticoids and the maintenance of optimal glycemic control, underscoring the importance of a multidisciplinary and integrated treatment approach. Further long-term studies are required to clarify the pathophysiological mechanisms of TINU syndrome in the context of chronic comorbidities, to better define the role of immunosuppressive agents, and to improve long-term renal and ocular outcomes.

## Data Availability

The original contributions presented in the study are included in the article/Supplementary material, further inquiries can be directed to the corresponding authors.

## Supplementary material

Supplementary Figure 1 Diagnostic and classification criteria for tubulointerstitial nephritis and uveitis syndrome.
